# A Low-Therapeutic Dose of Lithium Inhibits GSK3 and Enhances Myoblast Fusion in C2C12 Cells

**DOI:** 10.3390/cells8111340

**Published:** 2019-10-29

**Authors:** Nigel Kurgan, Kennedy C. Whitley, Lucas A. Maddalena, Fereshteh Moradi, Joshua Stoikos, Sophie I. Hamstra, Elizabeth A. Rubie, Megha Kumar, Brian D. Roy, James R. Woodgett, Jeffrey A. Stuart, Val A. Fajardo

**Affiliations:** 1 Department of Kinesiology, Brock University 1812 Sir Isaac Brock Way, St. Catharines, ON L2S 3A1, Canada; nk10dy@brocku.ca (N.K.); kw15wi@brocku.ca (K.C.W.); js17up@brocku.ca (J.S.); sh14iu@brocku.ca (S.I.H.); broy@brocku.ca (B.D.R.); 2 Centre for Bone and Muscle Health, Brock University 1812 Sir Isaac Brock Way, St. Catharines, ON L2S 3A1, Canada; 3 Department of Biological Sciences, Brock University 1812 Sir Isaac Brock Way, St. Catharines, ON L2S 3A1, Canada; LAM207@MRC-CU.cam.ac.uk (L.A.M.); fm15ta@brocku.ca (F.M.); jstuart@brocku.ca (J.A.S.); 4 Lunenfeld-Tanenbaum Research Institute, Sinai Health System, 600 University Avenue, Toronto, ON M5G 1X5, Canada; rubie@lunenfeld.ca (E.A.R.); kumar@lunenfeld.ca (M.K.); woodgett@lunenfeld.ca (J.R.W.)

**Keywords:** lithium, glycogen synthase kinase 3, myoblast fusion

## Abstract

Glycogen synthase kinase 3 (GSK3) slows myogenic differentiation and myoblast fusion partly by inhibiting the Wnt/β-catenin signaling pathway. Lithium, a common medication for bipolar disorder, inhibits GSK3 via Mg^+^ competition and increased Ser21 (GSK3α) or Ser9 (GSK3β) phosphorylation, leading to enhanced myoblast fusion and myogenic differentiation. However, previous studies demonstrating the effect of lithium on GSK3 have used concentrations up to 10 mM, which greatly exceeds concentrations measured in the serum of patients being treated for bipolar disorder (0.5–1.2 mM). Here, we determined whether a low-therapeutic (0.5 mM) dose of lithium could promote myoblast fusion and myogenic differentiation in C2C12 cells. C2C12 myotubes differentiated for three days in media containing 0.5 mM lithium chloride (LiCl) had significantly higher GSK3β (ser9) and GSK3α (ser21) phosphorylation compared with control myotubes differentiated in the same media without LiCl (+2–2.5 fold, *p* < 0.05), a result associated with an increase in total β-catenin. To further demonstrate that 0.5 mM LiCl inhibited GSK3 activity, we also developed a novel GSK3-specific activity assay. Using this enzyme-linked spectrophotometric assay, we showed that 0.5 mM LiCl-treated myotubes had significantly reduced GSK3 activity (−86%, *p* < 0.001). Correspondingly, 0.5 mM LiCl treated myotubes had a higher myoblast fusion index compared with control (*p* < 0.001) and significantly higher levels of markers of myogenesis (myogenin, +3-fold, *p* < 0.001) and myogenic differentiation (myosin heavy chain, +10-fold, *p* < 0.001). These results indicate that a low-therapeutic dose of LiCl is sufficient to promote myoblast fusion and myogenic differentiation in muscle cells, which has implications for the treatment of several myopathic conditions.

## 1. Introduction

Disease or age related declines in muscle mass and/or function is associated with falls, fractures, frailty, poor quality of life, and disability [1,2,3,4], all while being a significant predictor of all-cause mortality [5]. Strategies to maintain skeletal muscle mass throughout the lifespan could lead to significant benefits to overall health. Skeletal muscle is a dynamic and plastic tissue responding to internal (e.g., biochemical) and/or external (e.g., biophysical) stimuli, and an increase in strength and/or size (e.g., hypertrophy) can result from nutritional supplementation and exercise training [6,7,8]. Myonuclear accretion, which is accomplished through myoblast fusion [9], appears to be critical for muscle hypertrophy [10,11], as described in the myonuclear domain theory [12,13]. Furthermore, myoblast fusion enables repair of damaged muscle [14], which is important for recovery from exercise or injury as well as for constant muscle degenerating diseases, such as muscular dystrophy [15,16].

Glycogen synthase kinase 3 (GSK3) is a well known inhibitor of muscle hypertrophy [17,18] and myoblast fusion [19,20] that regulates a number of myogenic signaling pathways, including the Wnt/β-catenin pathway [21]. Lithium, a commonly prescribed bipolar medication [22], has been shown to inhibit GSK3 [23] and augment myoblast fusion [19]. However, previous in vitro studies showing myoblast fusion have used high doses (e.g., 10 mM [19,20]) that would likely be toxic in humans [24,25]. In a clinical setting, serum lithium levels are tightly regulated between 0.5–1.2 mM to prevent toxicity-related side effects, which include renal, thyroid, and parathyroid dysfunction [25]. In this study, we sought to determine whether a non-toxic low therapeutic dose (0.5 mM) of lithium would be sufficient to inhibit GSK3, promote β-catenin accumulation, and augment myoblast fusion in hopes of demonstrating the potential use of lithium as a therapy for muscle wasting conditions. 

## 2. Materials and Methods

### 2.1. C2C12 Cells

To examine the effects of low dose (0.5 mM) lithium supplementation on myoblast fusion, we utilized immortalized C2C12 myoblasts. These cells were cultured in growth medium [Dulbecco’s modified Eagle’s medium (DMEM), D6429 Sigma, Oakville, ON, Canada] supplemented with 10% fetal bovine serum (Sigma F1051), 1% penicillin/streptomycin (Sigma P4333), and 2% non-essential amino acids (Sigma M7145) at 37 °C in a humidified atmosphere with 5% CO_2_ and 5% O_2_ levels [26]. Once the myoblasts were at 80% confluence, the growth medium was replaced with differentiation medium (DMEM supplemented with 1% adult horse serum, 1% penicillin/streptomycin, 2% non-essential amino acids) that was either supplemented with 0.5 mM lithium chloride (LiCl group) or not (control group). The differentiation media with and without 0.5 mM LiCl was replenished every other day until day 3 of differentiation, where the cells were then scraped and pelleted for GSK3 activity assays and/or Western blotting. For GSK3 activity assays, the pelleted cells were lysed in assay buffer (250 mM sucrose, 5 mM HEPES, 0.2 mM PMSF, 0.2% [w/v] NaN_3_), whereas for Western blotting, the pelleted cells were lysed in RIPA buffer (20-188 Sigma Aldrich, MO, USA) supplemented with protease (cOmplete™, Roche, Upper Bavaria, Germany) and phosphatase inhibitors (PhosSTOP, Roche). All cell lysates were stored at −80 °C until further analysis.

### 2.2. Western Blotting

Western blotting was performed to determine the expression levels of phosphorylated GSK3β (ser9; 9336, Cell Signaling Technology, Beverly, MA, USA), total GSK3β (9315, Cell Signaling Technology), phosphorylated GSK3α (8542, Cell Signaling Technology), total GSK3α (4818, Cell Signaling Technology), total β-catenin (9582, Cell Signaling Technology), myogenin (F5D, Developmental Studies Hybridoma Bank, Iowa City, IA, USA) and pan-myosin heavy chain (MHC) (MF20, Developmental Studies Hybridoma Bank). Solubilized proteins from tissue homogenates and cell lysates were separated using 4–15% TGX BioRad precast gels and then transferred onto 0.2 μm polyvinylidene difluoride (PVDF) membranes. Subsequently, membranes were blocked with 3% bovine serum albumin in tris-buffered saline tween solution (TBS-T) and then immunoprobed with their corresponding primary antibodies. Membranes were then washed in TBS-T and immunoprobed with horseradish peroxidase-conjugated secondary antibodies. Chemiluminescent substrates, Immobilon (Millipore, Burlington, MA, USA), or Clarity Max (Bio-Rad, Hercules, CA, USA) were used to detect antigen–antibody complexes and were visualized using a BioRad ChemiDoc imager. Quantification of optical densities was performed using ImageLab (BioRad, USA) and normalized to total protein visualized using Ponceau staining. 

### 2.3. GSK3 Activity Assay

An enzyme-linked assay that measures ATP hydrolysis indirectly through NADH oxidation was developed and adapted for a 96-well plate format. Briefly, cell lysates were added to 100 ul of reaction buffer containing 200 mM KCl, 20 mM HEPES, 15 mM MgCl2, 10 mM NaN3, 10 mM phosphoenolpyruvate, 5 mM ATP, 1 mM EGTA, 18 U/mL lactate dehydrogenase, 18 U/mL pyruvate kinase, 0.3 mM NADH, 5 mM ATP, and 5 µg of a GSK3-specific substrate peptide [YRRAAVPPSPSLSRHSSPHQ(pS)EDEEE, G50-58 SignalChem, Richmond, BC, Canada], pH 7.0. Total ATP hydrolysis was inferred from the rate of NADH disappearance over 30 min measured at 37 °C and 340 nm using a spectrophotometric plate reader (M2 Multimode Reader, Molecular Devices). The concentration of NADH was calculated using the molar extinction coefficient 6.22 mM^−1^·cm^−1^ and was indicative of ATP hydrolysis in a 1:1 ratio. All experiments were performed in duplicate. GSK3-specific activity was measured using two approaches. First, we measured ATP hydrolysis in the presence and the absence of the GSK3 substrate, and GSK3 specific activity was calculated as the subtracted difference in the rates of ATP hydrolysis. Second, ATP hydrolysis was measured using the GSK3 substrate in cells incubated with a potent GSK3 inhibitor (CHIR99021, Sigma SML1046; 25 µM) or vehicle (DMSO). The GSK3 specific activity was then calculated as the difference between the rates of ATP hydrolysis in vehicle treated lysates and CHIR99021 treated lysates.

### 2.4. Generation of GSK3 Double-Knockout Cell-Line

DLD1 was obtained from American Type Culture Collection (ATCC, Manassas, VA, USA) and authenticated using STR (short-tandem repeats) profiling (The Centre for Applied Genomics, Hospital for Sick Children). Cells were cultured in Dulbecco’s modified Eagle’s medium (ThermoFisher Scientific, Waltham, MA, USA) supplemented with 10% fetal bovine serum (Wisent Bioproducts, Saint-Jean-Baptiste, QC, Canada) and penicillin-streptomycin (100 U/mL; ThermoFisher Scientific). For high efficiency clone selection, single guide (gRNAs) targeting different regions of exons 2 of each GSK3 isoform were cloned into separate all-in-one cas9-reporter vectors (Sigma-Aldrich) (Table 1) with a U6 promoter driving the expression of gRNA and a CMV promoter driving expression of CAS9-T2A-GFP (p01) or -RFP (p02) protein cassette. Cells were transfected using lipofectamine 3000 (ThermoFisher Scientific) and allowed to recover for 48 h prior to single-cell sorting (Astrios EQ, Lunenfeld Tanenabum Research Institute) based on green fluorescent protein (GFP) or red fluorescent protein (RFP) signal into 96-well plates for subsequent expansion. Single knockout cells were re-transfected with the appropriate second plasmid and single-cell sorted to generate double knockout cells. All clones were verified by immunoblotting and DNA sequencing.

### 2.5. Myoblast Fusion

Myoblast fusion assays were performed as previously described [27]. Briefly, 1 × 10^5^ myoblasts were seeded onto 35 mm poly-d-lysine coated petri dishes (P35GC-1.5-14-C, MatTek, Ashland, MA. USA) and incubated at 37 °C in a humidified atmosphere with 5% CO_2_ and 5% O_2_ levels in growth medium for 24 h. Subsequently, the growth medium was replaced with differentiation medium with or without 0.5 mM LiCl, which was replenished every other day until day 3. The day 3 differentiatied myotubes were then fixed by incubating them in a Triton-X supplemented formalin solution (0.5% Triton-X [v/v] in 10% neutral buffered formalin). Next, the fixed cells were blocked in a phosphate-buffered saline and 0.1% Tween (PBS-T) solution supplemented with 10% normal goat serum (G9023; Sigma-Aldrich) for 1 h. After blocking, the cells were incubated overnight with MHC IIa primary antibody (SC-71, Developmental Hybridoma Bank). On the next day, the cells were then probed with Alexa Fluor 488 IgG_1_ (A-21121, ThermoFisher Scientific) secondary antibody for 1 h, followed by a 4′,6-diamidino-2-phenylindole (DAPI) stain (D1306, ThermoFisher Scientific) for 2 min to stain the nuclei. A Zeiss Axio Observer Researcher Fluorescent Microscope and a digital camera (ORCA-Flash4.0 V2 Digital CMOS camera; C11440-22CU; Hamamatsu Photonics, Hamamatsu City, Shizuoka, Japan) were then used to visualize the myotubes at 20× magnification. For all analyses, 2–3 images per well (i.e., experimental n) were taken at random sections, and the total nuclei were counted using imageJ (National Institutes of Health, Bethesda, MD, USA). The fusion index was then calculated by dividing the number of nuclei in myotubes (myosin-positive with at least 2 nuclei) by the total number of nuclei analyzed (207–566 nuclei) as previously described [27].

### 2.6. Statistical Analysis

Results are expressed as mean ± SEM of each group. A Student’s unpaired T-test (two-tailed) was used to compare control and LiCl treated cells in all experiments. Sample size (n) refers to technical replicates for each individual experiment that were performed on at least 3 different passages (2–10). Statistical significance was assumed at *p* ≤ 0.05, and all statistical analyses were performed using Graphpad Prism 7 software. 

## 3. Results

### 3.1. A Low-Therapeutic Dose of LiCl Inhibits GSK3β and Total GSK3 Activity

**Figure 1:**
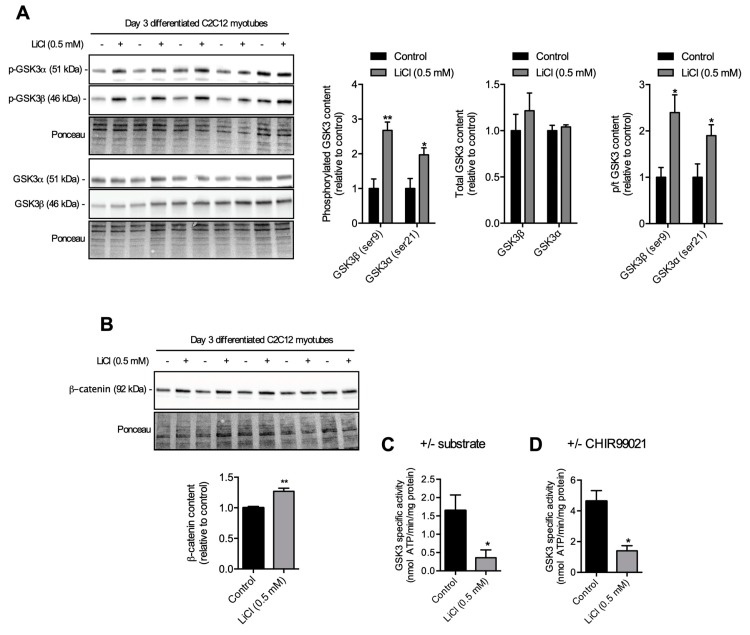
The effect of a low therapeutic dose of lithium on GSK3 serine phosphorylation, β-catenin content, and GSK3 activity. (**A**) A low therapeutic dose (0.5 mM) of LiCl had no effect on total GSK3 content but increased phosphorylation at ser9 (GSK3β) and ser21 (GSK3α) in day 3 differentiated C2C12 myotubes. (**B**) β-catenin content increased in cells treated with a low therapeutic dose (0.5 mM) of LiCl compared to non-treated cells (control). (**C**,**D**) Treatment of cells with a low therapeutic dose of LiCl (0.5 mM) had less GSK3 activity when assessed either in the presence or the absence of a GSK3 specific substrate (**C**) or a GSK3 specific inhibitor (**D**, CHIR99021, 25 µM). Significant difference from control using a independent Student’s t test, *p < 0.05; **p <0.01 (n = 6 per group).

The phosphorylation status of GSK3β and GSK3α on ser9 and ser21, respectively, can act as a surrogate marker of GSK3 inhibition. Figure 1A compares total GSK3 content and its serine phosphorylation status in cells treated with or without 0.5 mM LiCl. LiCl treatment led to a significant increase in phosphorylated GSK3β and GSK3α with no change in total GSK3 content compared to non-treated cells, which led to an overall increase in the ratio of phosphorylated to total GSK3. One function of GSK3 is to phosphorylate β-catenin, which marks it for degradation. Since both GSK3 isoforms appeared to be inhibited with increased ser phosphorylation, we hypothesized that there should also be an increase in total β-catenin content, which was observed (Figure 1B).

To determine directly whether GSK3 was inhibited, we developed a GSK3 specific activity assay. Figure S1A shows a linear relationship between GSK3 activity (ATP hydrolysis) with addition of increasing amounts (ng) of purified GSK3β protein (Promega, V1991, Madison, WI, USA), suggesting adequate sensitivity for changes in GSK3 activity. To examine GSK3-specific activity, we assessed the rates of ATP hydrolysis in the presence and the absence of the GSK3-specific peptide substrate. This assay revealed an approximately 85% reduction in GSK3 activity in LiCl-treated myotubes compared with controls (Figure 1C). To confirm the specificity of GSK3 for the substrate and to validate our approach, we analyzed GSK3-specific activity in wild-type (WT) and double *GSK3* knockout (GSK3^-/-^) DLD-1 cells (Figure S1B). As expected, GSK3^-/-^ cells showed no GSK3 substrate-dependent ATP hydrolysis, while ATP hydrolysis was stimulated by GSK3 substrate in WT cells. To further validate our assay, we next examined GSK3-specific activity in soleus and extensor digitorum longus (EDL) and found that EDL had a significantly lower (−63%) GSK3 activity than that found in the soleus (Figure S1C). Corresponding well with this, the soleus muscle had significantly higher total GSK3β content with relatively lower ser9 phosphorylation, which translated to a significantly lower (~35%) ser9p/total GSK3β ratio. Altogether, these findings demonstrate that our approach of assessing GSK3-specific activity is valid and is sensitive to changes in GSK3 activity. Finally, we also assessed GSK3-specific activity by measuring rates of ATP hydrolysis in the presence and the absence of a selective and potent GSK3 inhibitor (CHIR99021, 25 µM), and our results also demonstrate that LiCl-treated myotubes had significantly lower GSK3 activity (Figure 1D).

### 3.2. Sub-Therapeutic Dose of LiCl Augments Myoblast Fusion

**Figure 2:**
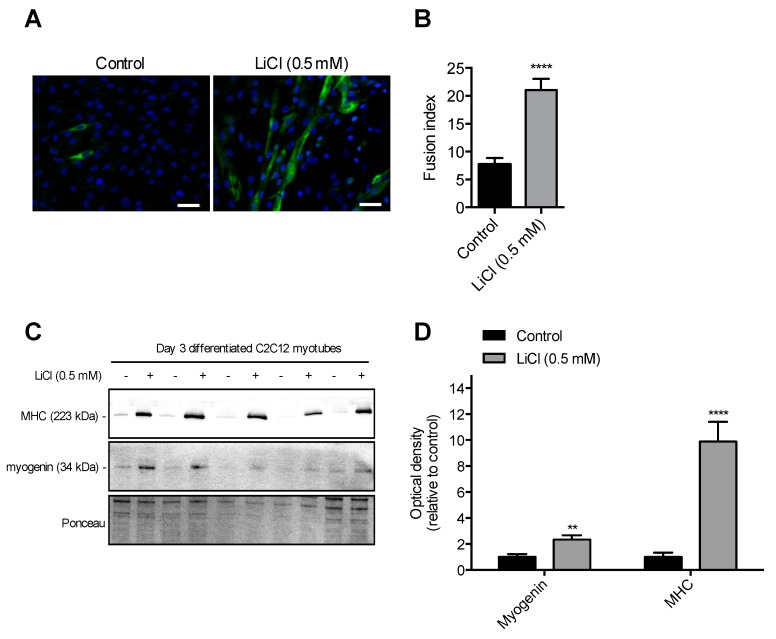
A low therapeutic (0.5 mM) dose of LiCl enhances myoblast fusion and muscle differentiation in C2C12 myotubes. (**A**) Representative images of day 3 differentiated C2C12 myotubes stained with 4′,6-diamidino-2-phenylindole (DAPI) for nuclei (blue) and myosin heavy chain IIa (MHC IIa, green). (**B**) Fusion index as calculated by the number of nuclei in MHC-positive myotubes containing at least two nuclei relative to the total nuclei. (**C**,**D**) MHC (pan-MHC) and myogenin content in low dose lithium treated and control treated cells assessed through Western blotting. (**B**) and (**C**) Significantly different from control using a independent Student’s t test, **p < 0.01; ****p < 0.001 (n = 6 per group).

Next, we examined the effect of 0.5 mM LiCl treatment on myoblast fusion and myogenic differentiation. C2C12 myoblasts treated with 0.5 mM LiCl had a 2.9-fold higher fusion index (*p* < 0.01) compared to control (Figure 2A,B). Levels of specific markers of muscle differentiation, myogenin and MHC, were 3- and 10-fold higher, respectively, in C2C12 cells treated with 0.5 mM LiCl compared to controls (Figure 1C,D). Taken together, these results indicate that 0.5 mM LiCl treatment increased myoblast fusion and differentiation.

## 4. Discussion

It is important to determine whether a therapeutic/non-harmful dose of lithium is sufficient to inhibit GSK3 and augment myoblast fusion. The therapeutic range in serum of bi-polar patients prescribed lithium is ~0.5–1.2 mM, which is tightly regulated to prevent the toxicity [25] observed at concentrations in the range of ~1.5–3.5 mM [22,28]. Even this toxic concentration is ~2–10× lower than the concentration typically used to study the inhibition of GSK3 in vitro, raising questions about the effectiveness of therapeutic lithium on GSK3 inhibition. Here, we showed that a low-therapeutic dose (0.5 mM) of lithium in the form of LiCl is sufficient to increase GSK3β (ser9) and GSK3α (ser21) phosporylation while correspondingly reducing GSK3 activity and increasing total β-catenin content. Concomitantly with inhibition of GSK3, we found a significant increase in myoblast fusion index as well as markers of myogenic differentiation (myogenin and MHC) in C2C12 cells treated with 0.5 mM LiCl. These findings confirm that therapeutic LiCl concentrations can cause GSK3 inhibition, as had previously been observed at doses above 10 mM [19,20,29]. 

In the present study, we show that 0.5 mM LiCl treatment inhibits both GSK3α and GSK3β via elevated serine phosphorylation. This could suggest that the effects of low dose lithium supplementation on myoblast fusion and myogenic differentiation may be mediated by both GSK3 isoforms. Indeed, GSK3β and GSK3α have been shown to be functionally redundant with respect to inhibiting Wnt/β-catenin signaling [30]. However, given that GSK3β has significantly higher expression levels than GSK3α in mammalian skeletal muscle [31], it is likely that GSK3β inhibition is most critical in mediating myoblast fusion. Similarly, GSK3β is the major regulator of glycogen synthase in murine skeletal muscle given that its expression is relatively higher than GSK3α [32]. This is likely why most studies addressing the role of GSK3 in regulating myoblast fusion and myogenic differentiation have focused on GSK3β rather than GSK3α [19,32,33]. In fact, knocking down GSK3β has been shown to augment myoblast fusion, perhaps suggesting that GSK3α cannot compensate and inhibit myoblast fusion in muscle [19,32,33]. Thus, although GSK3α was also inhibited under these conditions, it should be noted that GSK3β specifically is implicated in myoblast fusion and myogenic differentiation.

Our results are important for the clinical use of lithium. In vivo models of muscle wasting diseases such as muscular dystrophy [34], myotonic dystrophy [35] or age-related sarcopenia [36] may benefit from lithium treatment given the role of GSK3 in these diseases. To date, there is only one study [29] examining the impact of LiCl supplementation in a model of muscle wasting. Using a mouse model of limb girdle muscular dystrophy, the authors demonstrated that introduction of LiCl in the diet at a dose known to result in a steady-state serum lithium concentration of 0.5 mM (250 mg/kg/day) improved muscle size and strength [29,37,38]. Specifically, Findlay et al. [29] showed that mutations in the *DNAJB6* gene, which are known to cause limb girdle muscular dystrophy, also results in enhanced GSK3 activation and reduced β-catenin signaling in the affected skeletal muscles. Thus, the results from our present study showing that 0.5 mM LiCl can inhibit GSK3 activity, increase β-catenin levels, and augment myoblast fusion may provide additional mechanistic insight. 

In addition to its effects on GSK3, there are potentially some GSK3-independent effects of lithium that could benefit muscle health and function. For example, lithium has been shown to augment autophagy by regulating inositol triphosphate metabolism [39,40], and regular autophagic clearance is known to be critical for muscle strength and mass [41,42]. Furthermore, by activating phosphatidylinositide 3 kinase, lithium can augment mTOR signaling, which can enhance protein synthesis and muscle hypertrophy along with myoblast fusion. Therefore, our findings demonstrating the effects of 0.5 mM lithium treatment on augmenting myoblast fusion via GSK3 inhibition should be extended with future studies that explore other GSK3-independent pathways that may also promote muscle mass and strength. 

Aside from our findings demonstrating the therapeutic potential of low-dose lithium supplementation, we also developed a novel enzyme-linked GSK3 activity assay and validated it using wild-type and GSK3-null cell lysates. The assay can be used to measure GSK3 activity in purified GSK3, crude cell lysates, and muscle homogenates. It is based on the principals of our Ca^2+^-dependent sarco(endo)plasmic reticulum Ca^2+^-ATPase (SERCA) activity assay, with which we successfully measured SERCA activity in muscle homogenates and cell lysates (see references [43,44,45,46,47,48,49,50,51,52]). In this latter assay, SERCA-mediated ATP hydrolysis leads to phosphoenolpyruvate conversion to pyruvate via pyruvate kinase and subsequent conversion of pyruvate to lactate via lactate dehydrogenase (LDH). NADH oxidation by LDH is measured directly. In our GSK3 assay, GSK3-mediated ATP hydrolysis is linked to the same sequence of events and is similarly reported as NADH oxidation. The specificity of the assay is ensured by using a GSK3-specific peptide substrate, and indeed, we showed no activity in GSK3-null samples. 

When comparing between muscle types, maximal rates of SERCA activity are known to be greater in the EDL compared with the soleus [53] owing to the fact that the EDL muscles contain far more SERCA pumps [54]. Conversely, and with respect to GSK3, the soleus muscle contains more total GSK3 and less serine-phosphorylated GSK3 compared with the EDL (Figure S1E). In turn, our measurements of GSK3 activity using the GSK3 specific substrate demonstrate that the soleus muscle exhibits siginificantly greater GSK3 activity (Figure S1E). Also, the rates of GSK3 activity are at least 25–100× lower than the maximal rates of SERCA activity in murine skeletal muscle. This is to be expected. SERCA pumps contribute 50% of resting muscle metabolic rate [54], while GSK3 is far less metabolically active. Thus, our present results also reveal a novel method in detecting changes in GSK3 activity sensitive to pharmacological inhibition and alterations in GSK3 expression.

In summary, our study shows that a low therapeutic dose of lithium can inhibit GSK3 and augment myoblast fusion. These results suggest that a non-toxic therapeutic dose of lithium might be an effective option for promoting muscle development in vivo. Ultimately, this work will aid in determining whether Food and Drug Administration (FDA)-approved lithium can be used at clinically safe doses to enhance muscle hypertrophy and repair via GSK3 inhibition, potentially attenuating some of the muscle atrophy observed in different conditions. Since the development of novel selective and potent GSK3 inhibitors is an active field of research, our work revealing a novel GSK3 activity assay could also be useful in the validation of such inhibitors.

## Figures and Tables

**Table 1:** Cas9 reporter vectors for *gsk3α* and *gsk3β*.

Gene	Vector	Target Site	Targeted Region
*GSK3* *α*	U6gRNA-Cas9-2A-RFP	GCGCGGACTAGCTCGTTCGCGG	168–189
*GSK3β*	U6gRNA-Cas9-2A-GFP	GGCTTGCAGCTCTCCGCAAAGG	450–470

## Supplementary Materials

The following are available online at https://www.mdpi.com/2073-4409/8/11/1340/s1. Figure S1. Measuring GSK3 activity in purified GSK3, GSK3-null, and soleus and extensor digitorum longus (EDL) muscle samples.
