# Supplementary material for: A Low-Therapeutic Dose of Lithium Inhibits GSK3 and Enhances Myoblast Fusion in C2C12 Cells
Source: Cells. 2019 Oct 29;8(11):1340. doi: 10.3390/cells8111340 (PMC6912290; doi:10.3390/cells8111340)
Supplement: Supplementary file 1 [file cells-08-01340-s001.pdf]

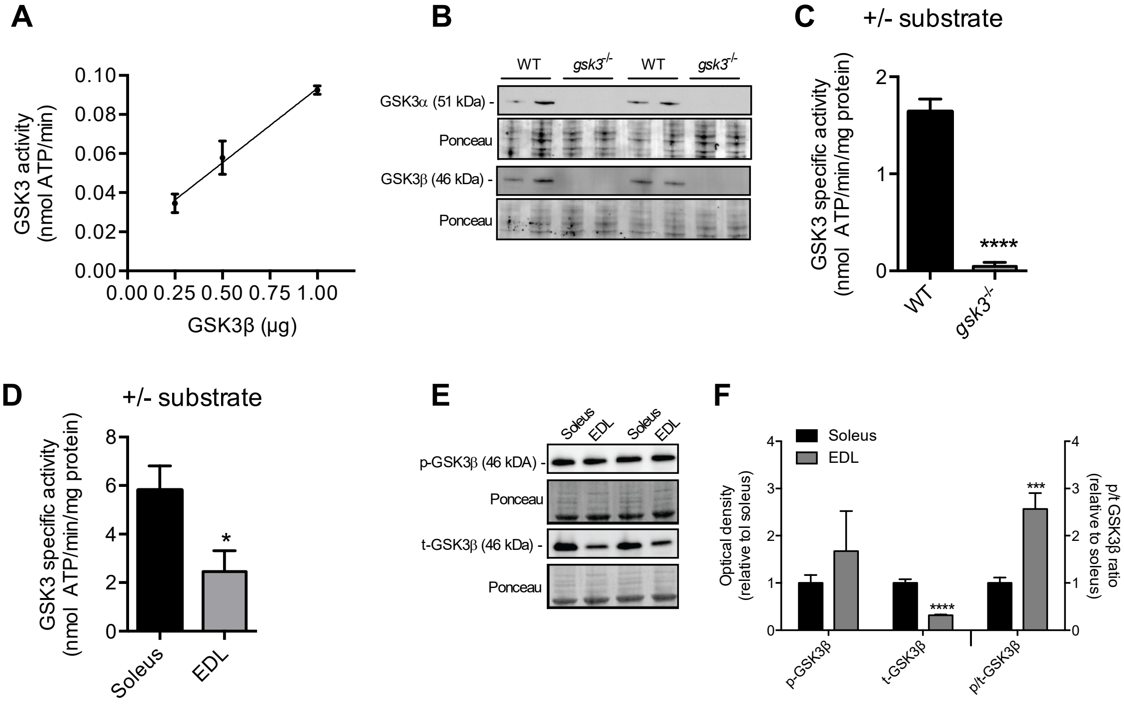

Figure S1. Measuring GSK3 activity in purified GSK3, GSK3-null, and soleus and extensor digitorum longus (EDL) muscle samples. A) Positive linear relationship between increasing amounts of purified GSK3β and GSK3 activity performed in quadruplicates. B) Representative Western blot images demonstrating successful deletion of *GSK3α* and *GSK3β* in DLD1 cells. C) GSK3 specific activity of wild type (WT) and gsk3-null DLD1 cells ($GSK3^{-/-}$) in nmol ATP/min/mg protein. D) Comparison of GSK3 specific activity of soleus and extensor digitorum longus (EDL). (E and F) Representative Western blot images and densitometric analyses of total GSK3β and serine 9 phosphorylation status in soleus and EDL. For (C), **** significantly different from WT using an independent Student's *t*-test, $p < 0.0001$ (n = 4 per group). For (D) and (E), significantly different from soleus using an independent Student's t-test, * $p < 0.05$, *** $p < 0.001$, **** $p < 0.0001$ (n = 6 per group).
